# Stroke Survivors' Experiences of Sleep: A Hermeneutic Phenomenological Study

**DOI:** 10.1155/nrp/1399026

**Published:** 2025-08-08

**Authors:** Ida Kathrine Westh, Louise Abildgaard Moeller, Marit Otto, Pia Dreyer, Grethe Andersen, Janne Kaergaard Mortensen

**Affiliations:** ^1^Department of Neurology, Aarhus University Hospital, Aarhus, Denmark; ^2^Department of Public Health, Aarhus University, Aarhus, Denmark; ^3^Intensive Care Unit, Aarhus University Hospital, Aarhus, Denmark; ^4^Department of Clinical Medicine, Aarhus University, Aarhus, Denmark

**Keywords:** interview, patient perspective, phenomenological-hermeneutic approach, rehabilitation, sleep disturbances, stroke

## Abstract

**Aim:** To gain new insights into the lived experience of poststroke sleep.

**Design:** Qualitative interview study.

**Methods:** The study was based on a phenomenological-hermeneutic approach inspired by the French Philosopher Paul Ricoeur. Fifteen participants were included between 2021 and 2022 and interviewed 3 months after stroke. Interviews were interpreted through naïve reading and structurally analyzed. Credibility was enhanced by investigator triangulation, and field notes were used for reflections during the interpretation process to challenge preconceptions.

**Results:** The participants experienced changes in sleep and described a fear of lying awake at night. They described a lack of energy and a heavy body and mind as well as an overwhelming sleepiness during daytime. Three themes were identified through the analyses: “Floating through the night—a mental state of mind being between awake and asleep,” “Left with a heavy and slow feeling—poor sleep leads to a lack of energy,” and “Sleeping during daytime may happen like lightning from a clear sky.”

**Conclusions:** The participants experienced frequent and substantial changes in sleep which affected their everyday lives in various ways.

**Summary**

• Implications
◦ Stroke survivors should be offered sleep interviews and professional information on poststroke sleep.
• Impact
◦ Stroke is associated with potential complications including sleep disturbances as found in this study.
◦ The findings of the study can inform health professional counseling and support the development of clinical guidelines as well as written materials for stroke survivors.
◦ Professional advice on sleep hygiene and awareness of potential concomitant mental poststroke complications that can contribute to sleep disturbances must be part of stroke survivors' rehabilitation.
◦ The findings can inspire researchers to further explore the interplay between sleep disturbances, anxiety, mood, and fatigue.
• Reporting method
◦ The Consolidated Criteria for Reporting Qualitative Research (COREQ) checklist was used for reporting this study.
• Patient contribution
◦ Fifteen stroke survivors were interviewed for this qualitative interview study.
• What does this paper contribute to the wider global clinical community:
◦ We highlight the importance of acknowledging sleep disturbances as an integral part of poststroke nursing care as well as the importance of giving relevant patient guidance on what to expect with regard to sleep after stroke.
◦ The paper contributes patient perspectives illustrating how patients may experience poor sleep, a lack of energy, and suddenly falling asleep.
◦ These insights may help guide healthcare professionals in their communication with patients after stroke.
◦ Finally, the paper draws attention to the potential interplay between sleep disturbances and concomitant poststroke mental complications such as anxiety, depression, and fatigue, which is important to recognize in both the clinical setting and in future studies.

## 1. Introduction

Despite the growing body of evidence showing that sleep disturbances are common after stroke [1–9], research on the patient perspective is scarce. To further explore the phenomenon of poststroke sleep, it is crucial to study the lived experience of poststroke sleep and to broaden our understanding.

The estimated annual stroke incidence is 12.2 million cases globally [10], and stroke is a leading cause of death and disability worldwide [11]. Stroke survivors are at a risk of several complications related to physical disability, cognition, communication, social lives, mood, fatigue, and activities of daily living. Poststroke sleep disorders further contribute to impaired functioning [12], and it is estimated that more than 50% of stroke survivors experience sleep disturbances [4]. Poststroke sleep disturbances occur as various forms of insomnia [2], sleep–wake cycle disturbances [4], sleep apnea, restless legs syndrome [1], and hypersomnia [12]. A sleep disturbance affects the short- and long-term recoveries and outcome of stroke rehabilitation [13, 14] and is an independent predictor of life satisfaction, as is stroke severity and depression [2]. For patients who are still of working age, returning to work has been identified as essential in terms of social functioning, quality of life, and wellbeing after stroke [15–17]. However, sleep disturbances and their impact on vitality may complicate returning to work.

Overall, poststroke sleep disturbances are common and greatly impact stroke outcomes.

Still, whereas former studies have focused on the prevalence and polysomnographic characteristics of different sleep disorders after stroke, qualitative research exploring the patient's perspective is widely missing. This contrasts with an ample qualitative literature on poststroke fatigue as a daytime symptom not necessarily related to poor sleep [18, 19]. In the present study, we therefor aim to explore the patient's perception of poststroke sleep using a qualitative approach to complement the already existing quantitative knowledge.

Understanding the patient's perspective may help to guide healthcare professionals in their communication with patients and caregivers after stroke. Furthermore, in-depth understanding of poststroke sleep is crucial to inform future studies and to potentially develop new interventions.

## 2. Materials and Methods

With the research question of how stroke survivors experience sleep, we conducted a qualitative interview study with the aim of gaining new insights into the lived experience of poststroke sleep.

### 2.1. Design

The study was a qualitative interview study.

### 2.2. Theoretical Framework

Using a phenomenological-hermeneutic approach, the focus is on patients' lived experiences. When interviewing, the interviewee strives for openness to capture the patient's first-hand perspective. Therefore, to gain in-depth understanding of poststroke sleep, the study draws on the phenomenological-hermeneutic methodology inspired by the French Philosopher Paul Ricoeur [20].

### 2.3. Study Setting and Recruitment

The study participants were recruited from the Rehabilitation Unit and the Outpatient Clinic at the Stroke Department at Aarhus University Hospital in Denmark from October to December 2021. Inclusion at the Rehabilitation Unit was undertaken through a face-to-face invitation by the first author working in the rehabilitation unit as a part time nurse. The participants included from the outpatient clinic were invited through a face-to-face invitation during the doctor's follow-up appointments (author GA). Both the interviewer, as a nurse at the Rehabilitation Unit, and the doctor in the outpatient clinic had engaged in routine poststroke care and relevant medical treatment before recruitment. Participants were informed that the first author conducted the study as part of her academic training. Some participants were included within days after their stroke, whereas others were included after several weeks. All participants, however, were interviewed 3 months after stroke onset.

#### 2.3.1. Stroke-Specific Information

Information on stroke severity at admission was measured by the National Institute of Health Stroke Scale (NIHSS) as part of the routine stroke admission and registered in the patients' medical records. The NIHSS score ranges from 0 to 42 with a higher score indicating more severe stroke symptoms [21]. Information on stroke location was obtained from imaging descriptions.

### 2.4. Inclusion and Exclusion Criteria

Inclusion criteria were as follows:
    Patients with imaging verified ischemic or hemorrhagic stroke, all ages
    Admitted to the rehabilitation unit or at the outpatient clinic
    Able to give signed, informed consent
    Able to be interviewed in Danish 3 months after the acute stroke
Exclusion criteria were as follows:
    A registered diagnosis of depression in the patients' medical records, antidepressant treatment
    Current or prestroke treatment with sleep sedatives

#### 2.4.1. Sampling Strategy

The participants were sampled using a purposive sampling strategy, which implied reflections on which circumstances or factors may cause variation in the experience of poststroke sleep. Purposive sampling is meaningful in qualitative interview studies to ensure a broader perspective on the phenomenon [22]. The following factors were considered to have an impact on the experience of the phenomenon: stroke location, gender, age, occupational status, and living arrangements (whether they lived alone or with a spouse or children). Information on NIHSS was collected from patients' medical records and registered to get a scientific overview of the stroke severity of the participants in this study, but participants were not specifically sampled according to the NIHSS score.

Former studies have shown that stroke location is correlated to sleep disturbances [23]. Gender and age were considered because we wished to make findings as heterogenic as possible. Occupational status can influence sleep due to increased stress load, and living alone may cause anxiousness during the night. Living with children affects the possibility to rest during daytime and may cause disturbances during nighttime, potentially affecting their caretaker's sleep.

Data saturation was continuously discussed, and an awareness to include participants with new perspectives that could potentially broaden the reported experience of poststroke sleep was kept in mind. Thus, when a patient was eligible for inclusion, according to the inclusion and exclusion criteria, the above-mentioned characteristics of the candidate were discussed and compared to the characteristics of the already included patients. A discussion was then made whether to include or not.

The exclusion criteria were chosen as depression has a known influence on sleep, and the use of sleep sedatives would have biased our findings. Information on a registered diagnosis of depression, antidepressive treatment, and current or prestroke treatment with sleep sedatives was retrieved from the patients' medical records.

### 2.5. Data Collection

Data were obtained through 15 semistructured interviews conducted by the first author in 2021 and 2022. All participants as well as the first author spoke Danish as their primary language, and all interviews were conducted in Danish. The interviews were carried out 3 months after the stroke incident in the participants' homes and lasted approximately half an hour. Participants' relatives were present during the interviews if the participant requested their presence. An interview guide was developed to operationalize the research question: “*How do stroke survivors experience sleep?*” and was qualified and adjusted through a pilot interview. The adjusted guide contained open-ended questions such as “How is a good night's sleep described,” “What influences sleep,” “What is important regarding sleep,” and “How has sleep changed?” This was to help the interviewer probe the phenomenon of poststroke sleep. The experienced level of energy and how this affected the participants' ability to carry out daily activities were also explored. Repeat interviews were not conducted.

The interviews were tape-recorded and transcribed verbatim by the first author, and field notes were taken.

### 2.6. Data Analysis

Data were analyzed using a Ricoeur-inspired analysis method described by Dreyer and Pedersen [24]. This method focuses on moving from a surface interpretation—*the naïve reading*—to an in-depth interpretation through *structural analysis* and *critical discussion*. Ricoeur states that the surplus of meaning can be both the case stated, the meaning beyond a text, and the interpretations made from the reader [20]. Thus, dialectic movements between “what is said,” “the meaning of the said,” and themes identified in the interpretation entail a new comprehensive understanding. In this study, the dialectic movements between what was said by the participants and the meaning of the said were analyzed through the authors' reflections on every sentence and coming to an agreement on the meaning. The initial review was made by the first and second authors independently, before discussing them and creating the naïve reading and the structural analysis. The collected field notes were also included in this process and were used for reflections during the interpretation process to challenge preconceptions. The interpretation process was qualified in the critical discussion phase by the third author being subspecialized as a sleep specialist, the two stroke neurologists and the two neurological nurses, as well as a professor nurse in the author team. In this process, the preconceptions were challenged, and a comprehensive understanding was thereby the result of this investigator triangulation [25], with in-depth analyses by the author team, using both physician expertise and nursing experience.

The interviews were translated into English after the structural analysis but before the critical discussion. Transcripts and results were not returned to the participants for comments.

### 2.7. Ethical Considerations

In accordance with the Danish law, the study did not require ethical approval from the local ethics committee, as it was approved as a quality assurance project. The project was registered in the regional quality project registry (755729), ensuring compliance with data protection regulations and approval by the Danish Data Protection Agency [26].

The participants gave written informed consent after receiving both written and oral participant information. The participants were anonymized with a number in the citation section. The study complies to the Declaration of Helsinki.

### 2.8. Rigor and Reflexivity

The COnsolidated criteria for REporting Qualitative research (COREQ) checklist was used for reporting this study.

The preliminary analyses were done by the first and second authors who coded the data material and continuously discussed the meaning of the coded quotations. The preconceptions of the first author were based on 20 years of stroke nursing experience and a master's degree in health science. This included the experience that stroke survivors need care concerning their sleep and that there is a general lack of interventions and knowledge to provide the necessary help. The first author has experience with qualitative interviews through her master's thesis process.

## 3. Findings

In this section, we describe the characteristics of the participants and the findings of the analyses and interpretation process. The findings are presented as themes. Selected quotations serve the purpose of creating transparency in the interpretation process.

### 3.1. Characteristics of Participants

The characteristics of the 15 participants are shown in Table 1. None of the included participants dropped out, and none of the invited participants declined to participate. The NIHSS score was between 0 and 6, indicating milder stroke symptoms within the sample. All included participants had ischemic stroke.

### 3.2. The Themes

#### 3.2.1. Floating Through the Night—A Mental State of Mind Being Between Awake and Asleep

The experience of not being able to fall asleep, despite the use of sleep-inducing strategies that were effective prestroke, could lead to a fear of never being able to sleep the way they used to. Strategies reported to promote sleep prior to the stroke incident were mindfulness, counting sheep, getting up to eat something, or drink hot milk. As these strategies were no longer effective, the nights were described as terrible to get through. Fear of the night approaching and fear of getting no sleep was described by a participant in the following:

*“Well, I've become afraid of sleeping. I think that all of this has shocked me so bad that I speculate too much when I lie in bed. That's why I stall time, before I go to bed. Then I might fall asleep after half an hour. If I go to bed too early, I will lie there for hours.” (i. 22)*

Experiences of interrupted and superficial sleep were common among the participants, and some of them even had a feeling of being in a state between awake and asleep throughout the night. Descriptions of a kind of floating experience that could go on during the entire night, resulting in an experience of only obtaining a level of superficial sleep, were described in many of the interviews. An ongoing experience of being just on the verge of awakening most of the night, just under the surface of becoming awake, was described by a participant like this: *“But you just lie there, floating…” (i. 15)*. He explained the feeling of thoughts wandering, not sleeping, and not being awake, like floating on the surface of the ocean.

The experience of sleeping just under the surface was also described as a feeling of having staring eyes. Like watching the clock hour by hour, getting only a bit of sleep but being somewhat awake under the surface. It was described as looking at the clock and simultaneously having the experience of floating in a mental state between being awake and asleep. One participant described the experience this way:

*“It's strange, because sometimes you feel like you don't sleep, you know? You have a feeling of seeing the clock change by the hour and yet, it feels like you're well rested in the morning. Something must have slept, right? But apparently not my gaze.” (i. 19)*

Nights with very little sleep were described:

*“I sleep 1–3 h every other night, and it happens that I sleep longer the other nights.” (i. 20) Interrupted sleep was also described as a problem: “It is to continue sleeping—that is the problem.” (i. 18).*

Lying there, not being able to fall asleep again, was described as difficult, and speculating became a problem. Further, waking up in the early morning made it difficult to fall asleep again:

*“Some nights, if I wake up, I just lie there speculating and I have a hard time falling asleep again. If I wake up late during the night, it's also hard for me to fall asleep again.” (i. 26)*

One participant described that falling asleep was specifically difficult after waking up between 3 and 4 a.m.:

*“There is a certain time where I just can't fall asleep again, and that's around 3 or 4 a.m.—way too early.” (i. 24)*

Some of the participants experienced dreaming in a new way. One participant described it like this:

*The sleep pattern has not changed; the only change is that I am dreaming. (i. 13)*

Another participant used the following description:

*“I dream about a lot of things. Sometimes I dream about things that happened way back or people I haven't seen for many years.” (i. 16)*

It seemed that the dreaming was part of early awakenings and falling asleep again for this participant:

*In the beginning I was very tired but it's better now; I sleep 45 mins during the day, and then I sleep ok at nighttime. Some nights I sleep the whole night, and sometimes I wake up at around 3. 30 a.m. and I lie awake for 1 h and then I fall back to sleep. And that's when I dream a lot. (i. 21)*

#### 3.2.2. Left With a Heavy and Slow Feeling—Poor Sleep Leads to a Lack of Energy

There was variation in the participants' experiences of the connection between feeling rested and experiencing a good night's sleep. A participant reflected on the variability in the feeling of rest: *“Some days I feel really tired when I wake up, even if I quickly fell asleep, and some days I feel rested.”* (i. 22). This statement illuminates the subjective nature of rest, suggesting that the mere act of falling asleep does not guarantee a restorative experience. Instead, it hints at a complex relationship where emotional and physical states intertwine, influencing the perception of rest. After a good night's sleep, an easy and calm feeling in the body and mind was described: *“I don't know if I feel more rested. I just feel calmer, because I've slept well last night.” (i. 21).* The impact of poor sleep was also shown in the participant's narratives. One participant expressed it like this: *“Then I don't feel like going for a walk, going shopping or making dinner. These days, I feel heavy.”* (i. 12). This heaviness may symbolize not just physical exhaustion but profound exhaustion that invades daily activities, illustrating how the experience of poor sleep may lead to a disconnection from life's simple pleasures. Poor sleep was also described as causing different bodily sensations, and by one participant it was described as being hit by a formerly unknown overwhelming sensation of exhaustion. Another participant described it as a feeling of being hit with a hammer in the head: *“It gets better, but in the beginning, when I couldn't even do the laundry—It really hits you like a hammer in the head” (i. 23).* This intuitive imagery conveys the intensity of exhaustion, suggesting that poor sleep can lead to an acute awareness of one's limitations.

An empty feeling was described continuously in the data in relation to not getting enough sleep. The empty feeling could emerge as an experience of disconnection from the surroundings or one's former abilities. One participant described: *“Something happens in my head. I disconnect. I can't interact” (i. 13).* Another participant described: *“I get so slow. Like you haven't gotten enough sleep. It gets harder to concentrate. Well, I guess I get annoyed more easily, when I'm not well rested.” (i. 22)* This disconnection reflects changes in how to engage with activities in daily life and highlights how sleep disturbances may affect one's sense of self and agency.

#### 3.2.3. Sleeping During Daytime May Happen Like Lightning From a Clear Sky

In the interviews, daytime sleep was described as a sudden and unexpected experience, reported by one participant as “lightning from a clear sky.” Participants expressed a newfound and often overwhelming urge to rest during the day. One participant stated: *“I take a nap for half an hour between noon and 1 p.m., and then again at 6:30 p.m.*” (i. 15). This quote not only illustrates an adaptation to new needs but also reflects an acceptance that rest has become an integral part of daily routine. Recommendations to incorporate naps in the rehabilitation process were described. A participant notes: “*I was advised to rest for one or 2 h during the day, so I do that from 1:30 to 3:30 p.m. Sometimes I sleep for 15 min, sometimes for 45 min*” (i. 21). Another participant reported a necessary change in the daily routine due to the need for rest: *“An hour's rest at noon—I have never done that before but now I have to”* (i. 16). This highlights the need to incorporate rest into daily habits, and not as a luxury but a necessity. Moreover, some participants experienced falling asleep easier and faster than before. One participant articulated: *“I fall asleep much easier than I did before. If I lie down, I will certainly fall asleep*” (i. 18). This can be interpreted as a deeper connection to bodily needs, where it is now impossible to ignore the need for rest. Finally, participants described instances where sleep occurred unexpectedly, even in situations where it was not intended. “*It may also happen in the morning when I sit and relax. I'm not always asleep, but it can occur like lightning from a clear sky*” (i. 19). This statement underscores the spontaneous and unforeseen nature of sleep, which can overwhelm during moments of relaxation. It illustrates how the need to sleep has become an uninvited guest in daily life, emphasizing a new poststroke reality.

## 4. Discussion

### 4.1. Strengths and Limitations

We found the chosen methodology and theoretical framework suitable for answering our research question as it enabled us to account for the particularly complex interplay of physical symptoms with social and environmental factors among patients with stroke. The methodology allowed us to reveal insights beyond the physiological aspects of poststroke sleep by adding information on the personal experiences of the patients as to how they experienced sleep after stroke, what meaning they ascribed to these experiences, and how it affected their quality of life, recovery, and sense of well-being. The interpretation of the data and identification of themes allow for an empathetic understanding of poststroke sleep focusing on patient-centered care.

Former studies have described a journey after stroke toward a new self-image [27–29]. The participants in this study were likely also on a pathway of learning to cope with potential poststroke deficits, concerns of other diseases, and the fear of new strokes [30]. For some of the participants, further medical investigations also continued over a longer period, which could have influenced sleep and reinforced the experience of exhaustion. Being on this journey could not only have affected their sleep but could also have affected the way they thought and felt about their sleep and how they reported these thoughts and feelings to the interviewer.

The study was conducted while the first author was a member of the stroke care team at the Rehabilitation Unit. The researcher's extensive knowledge of stroke care, and the frequent interaction with patients with stroke, could have been an advantage and a disadvantage in the search of understanding the phenomenon of poststroke sleep. On the one hand, it facilitated the understanding of the underlying medical issues and the care needs of the participants. On the other hand, giving nursing advice easily became part of the interviews, which resulted in passages that did not enlighten the experience of poststroke sleep, but instead centered on the participants seeking nursing advice. Thus, during the interviews, participants often asked why poststroke sleep disturbances occur and how to overcome them. This illustrated the need for research in this area, and the interviewer responded by repeating the rationale of the study. Advice was given, according to standard sleep recommendations and from the interviewer's own nursing experiences, after performing the interview.

The researcher's preexisting knowledge on stroke nursing care could have caused blind spots, which could have hindered new understandings. The interviewer sought to overcome this by keeping an open mind during the interviews and staying aware of her preconceptions, both through reflections with the second author, and through field notes. The purpose of the notes was to reflect on the interviewer's role during the process and to make initial assumptions of what was said behind, and between, the spoken words in the interviews.

Although an effort was made to include a heterogeneous group of participants, the mild severity of strokes and lack of participants with a hemorrhagic stroke may limit the transferability of our findings.

Aphasia, dysarthria, high age, strong dialect, and psychological crises made it difficult to quote some of the participants' verbatim. Furthermore, the Danish version was translated into English after the structural but before the critical analysis. Overall, this could have led to bias, and we therefore strove to enlighten the meaning of the interviews. The findings thus became themes during the comprehensive understanding, both through analyzing the transcriptions and by incorporating the notes taken during the interviews.

Finally, the study has some built-in limitations. The focus of the research question was challenged, as experiences of sleep and fatigue interact. Also, low mood and anxiety could contribute to, or exaggerate, poststroke sleep disturbances and we cannot rule out that participants could have developed a depression by the time of the interview. Finally, we did not have information on potential sleep apnea.

### 4.2. Discussion of the Themes

In the first theme: *Floating through the night—a mental state of mind being between awake and asleep—*the floating experience and the feeling of sleeping superficially may be explained by sleep instability caused by many arousals. Sleep disruption with a feeling of superficial, unrefreshing sleep may not only correspond to an underlying new onset insomnia but may also be a symptom of pre- or poststroke sleep disorders such as sleep apnea or periodic limb movement disorder [31].

Repeated awakenings throughout the night were described as very distressing by the participants and the nights could feel like they were never ending. The fear of going to bed, due to a fear of not being able to sleep, was thus greatly distressing.

The participants were screened for the presence of a depression diagnosis through a review of their medical records, prior to consenting to participate in this study, but even so, some of them had symptoms of depression revealing during the interviews. Early awakening is a core symptom of poststroke depression, which occurs in approximately one-third of patients within 3 months, and anxiety is a common complication after stroke [32]. Some of the participants described early awakening, worries, and anxiety and may thus have had undiagnosed depression.

In the theme: *Left with a heavy and slow feeling—poor sleep leads to lack of energy,* participants described how poor sleep led to feelings of a heavy head and a slow feeling. This was experienced as disturbing the participants' everyday life, compromising their quality of life.

A paradox in the data was that some of the participants described how they felt rested after nights with experiences of superficial sleep. Short awakenings during normal sleep are forgotten the next day but after stroke, disturbances in sleep–wake circles are common [23]. A part of the explanation for this paradox could therefore be that the normal short awakening phases are a bit longer after stroke, which could result in a memory of the awakenings the next day. This may be interpreted as an abnormal interrupted sleep pattern but may not result in tiredness the next day.

Underlying sleep disturbances were probably present prestroke among some of the participants and may have worsened after stroke. A previous study showed that diary reports for total sleep time, number of wake periods, and daytime sleep were not different between a group of stroke patients and matched controls, although stroke patients reported significantly poorer sleep efficiency than controls [18].

In the theme: *Sleeping during daytime may happen like lightning from a clear sky,* the participants described how they suddenly fell asleep, and one patient described how it was experienced as lightning from a clear sky. Former studies have described daytime sleepiness poststroke [33], and this is probably what is being described among other experiences in this study. Whether patients describe a fatigue problem causing daytime sleepiness or if it is the other way round is not clear. Fatigue is a frequent complaint poststroke independent of sleep [34], but sleep disturbances may increase the fatigue severity.

### 4.3. Recommendations for Further Research

In further studies, it would be beneficial to distinguish between mood, fatigue, sleep disorders, and anxiety. Studies exploring the benefits of interventions to promote poststroke sleep are recommended.

### 4.4. Implications for Policy and Practice

Poor sleep can weaken the immune system, increase the risk of cardiovascular events [35], and reduce performance and the ability to learn [33, 36]. Further, some studies suggest that poor sleep can lead to a higher risk of Alzheimer's disease [37]. Awareness of sleep disorders poststroke is therefore very important. Patients may not be sufficiently rested to properly participate in rehabilitation and to cope with the challenges of living with deficits. Former studies suggest that quality of life is related to sleep problems [19], which we also found in our study. It is therefore highly recommended that screening for sleep disturbances should be part of poststroke rehabilitation programs. A systematic sleep interview should be mandatory after stroke to not only offer advice and education to stroke survivors but also identify potentially relevant patients for polysomnography and diagnosing sleep apnea.

## 5. Conclusion

The participants in this study experienced changes in sleep after stroke and described fear of long nights lying awake in bed, or a state of floating between awake and asleep and early morning awakenings. Nights with poor sleep resulted in lack of energy and was expressed as having a feeling of a heavy body and slow brain functioning. They also described an overwhelming sleepiness during daytime like lightning from a clear sky.

These insights into the lived experiences of sleep after stroke broaden our understanding and highlight the patients' need for guidance and information on poststroke sleep disturbances. This underlines that sleep disturbances should be part of poststroke rehabilitation programs to educate stroke survivors, caregivers, and to increase staff awareness. A systematic sleep interview should be mandatory after stroke to offer polysomnography if relevant, as well as screening for depression.

The phenomenon of poststroke sleep is complex, and objective measures of stroke sleep patterns should be considered to fully understand and treat sleep disturbances and unravel the interplay with mood, anxiety, and fatigue symptoms after stroke.

## Figures and Tables

**Table 1:** Socioeconomic characteristics, infarct location, age, and National Institute of Health Stroke Scale (NIHSS) of the participants.

Sex	Age	Infarct location	Acute NIHSS	Living alone	Living with children	Working
Female	74	Left subcortical hemisphere	0	+	−	−
Male	73	Right side of pons and right corona radiata	0	−	−	−
Male	64	Left medulla oblongata and cerebellum	4	−	−	+
Male	72	Left occipital lobe	2	+	−	−
Female	82	Left occipital lobe and right subcortical hemisphere	2	−	−	−
Male	73	Right thalamus and temporal and occipital lobes	2	−	−	−
Male	50	Right subcortical hemisphere	0	−	+	+
Female	77	Right side of pons	2	+	−	−
Male	61	Left medulla oblongata	1	+	−	+
Female	69	Left capsula interna	3	+	−	+
Male	62	Right thalamus	5	+	−	+
Female	59	Left corona radiata	1	−	−	+
Female	67	Left side of pons	6	−	−	−
Female	28	Right basal ganglia	2	−	−	+
Female	42	Left cortical and subcortical hemisphere	2	−	+	+

## Data Availability

Data are available from the REDCap prior to publishing.
